# A Single Gene Target of an ETS-Family Transcription Factor Determines Neuronal CO_2_-Chemosensitivity

**DOI:** 10.1371/journal.pone.0034014

**Published:** 2012-03-29

**Authors:** Julia P. Brandt, Sonya Aziz-Zaman, Vaida Juozaityte, Luis A. Martinez-Velazquez, Jakob Gramstrup Petersen, Roger Pocock, Niels Ringstad

**Affiliations:** 1 The Helen L. and Martin S. Kimmel Center for Biology and Medicine at the Skirball Institute of Biomolecular Medicine, Molecular Neurobiology Program, and Department of Cell Biology, NYU Langone Medical Center, New York, New York, United States of America; 2 Biotech Research and Innovation Centre (BRIC), University of Copenhagen, Copenhagen, Denmark; Harvard University, United States of America

## Abstract

Many animals possess neurons specialized for the detection of carbon dioxide (CO_2_), which acts as a cue to elicit behavioral responses and is also an internally generated product of respiration that regulates animal physiology. In many organisms how such neurons detect CO_2_ is poorly understood. We report here a mechanism that endows *C. elegans* neurons with the ability to detect CO_2_. The ETS-5 transcription factor is necessary for the specification of CO_2_-sensing BAG neurons. Expression of a single ETS-5 target gene, *gcy-9*, which encodes a receptor-type guanylate cyclase, is sufficient to bypass a requirement for *ets-5* in CO_2_-detection and transforms neurons into CO_2_-sensing neurons. Because ETS-5 and GCY-9 are members of gene families that are conserved between nematodes and vertebrates, a similar mechanism might act in the specification of CO_2_-sensing neurons in other phyla.

## Introduction

CO_2_-chemosensitive neurons are found in many animals. In vertebrates, CO_2_-sensing neurons are critical regulators of respiration [1]. Their dysfunction is proposed to underlie disorders such as sudden infant death syndrome [2] and congenital hypoventilation syndrome [3]. CO_2_ is also sensed by animals as an ethologically relevant environmental cue. For example, insects detect CO_2_ in the contexts of host- and mate-finding and as an aversive odorant [4], [5], and the rodent olfactory system contains neurons that can be activated by low concentrations of CO_2_ [6], [7]. Studies of the insect olfactory system have identified odorant receptors that mediate CO_2_ sensation, indicating that CO_2_ can act through cellular and molecular systems dedicated to its detection [4], [8], [9]. The molecular mechanisms that mediate CO_2_ sensing by insect olfactory neurons are, however, unique to insects. How neurons of other organisms detect CO_2_ is poorly understood.

To control internal concentrations of respiratory gases, the microscopic nematode *C. elegans* navigates to environments with preferred concentrations of oxygen and CO_2_ [10], [11], [12], [13], [14], [15]. Two anterior sensory neurons, the BAG neurons, detect environmental CO_2_ and mediate a CO_2_-avoidance behavior [15], [16], [17]. CO_2_-sensing by BAG neurons requires cyclic nucleotide signaling; mutants that lack either TAX-2 or TAX-4 subunits of a cyclic nucleotide-gated ion channel are defective in behavioral and physiological responses to CO_2_ [16], [17], as are mutants that lack the receptor-type guanylate cyclase GCY-9 [17]. Because CO_2_ activates the BAG neurons of *C. elegans* through a specific molecular pathway, their study offers the opportunity to understand the molecular basis of neuronal CO_2_-sensing.

ETS-5, an ETS-domain-containing transcription factor, was recently shown to regulate expression of many BAG-neuron-specific genes [18]. Whether ETS-5 is required for BAG-neuron responses to CO_2_, and, if so, how ETS-5 confers CO_2_-chemosensitivity to BAG neurons is unknown. We show here that *ets-5* mutants have defects in CO_2_ sensory transduction, and that ETS-5 directly interacts *in vitro* and *in vivo* with elements in the promoter of *gcy-9*, which encodes a critical component of the CO_2_ sensory transduction pathway. Strikingly, the requirement for ETS-5 in CO_2_ sensing can be bypassed by forced expression of *gcy-9*. These data indicate that a mechanism by which ETS-5 specifies CO_2_ chemosensitivity of BAG neurons is by direct regulation of the expression of GCY-9. GCY-9 in turn is sufficient to mediate neuronal responses to CO_2_ and likely encodes a receptor for CO_2_ or a CO_2_ metabolite.

## Results

### One of ten ETS-family transcription factors encoded by the *C. elegans* genome is required for the specification of CO_2_-sensing BAG neurons

**Figure 1:**
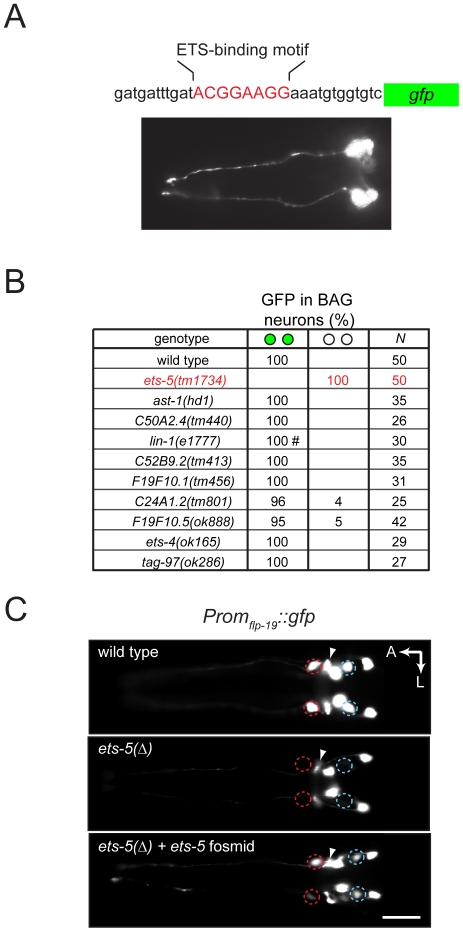
An ETS-family transcription factor is required for the specification of *C. elegans* CO_2_-chemosensitive BAG neurons. (A) A 31 basepair DNA element comprising a single ETS-binding motif (top) drives expression of GFP specifically in the BAG chemosensitive neurons (bottom). (B) One of ten ETS-family transcription factors encoded by the *C. elegans* genome is required for specification of BAG neurons. Shown is percent of animals mutant for each of ten ETS-family transcription factors encoded by the *C. elegans* genome that are BAGL/R ON (green circles) and BAGL/R OFF (open circles) for expression of a *Prom_flp-19_::gfp* reporter transgene. *N* = number of animals scored. # We found one *lin-1(e1777)* mutant in which *Prom_flp-19_::gfp* was not expressed in BAGR. (C) Fluorescence micrographs of *Prom_flp-19_::gfp* expression in a wild-type animal, an *ets-5* mutant and an *ets-5* mutant carrying a wild-type copy of the *ets-5* locus in a fosmid-derived transgene. BAGL/R neuron positions are marked by red circles and cells previously identified as AWAL/R [19] are marked by blue circles. The nerve ring is indicated by an arrowhead. The scale bar in lower panel is 20 µm. A: anterior, L: left. The *ets-5* mutant allele was *tm1734*. The *Prom_flp-19_::gfp* transgene was *ynIs34* and the *ets-5* rescuing transgene was *rpEx246*.

To identify factors that specify BAG neurons, we analyzed promoters of genes that are expressed by BAG neurons. We hypothesized that identification of cis-regulatory elements required for expression of these terminal differentiation genes in BAG neurons would enable us to identify trans-acting factors required for BAG neuron differentiation. First we looked at the promoter of the BAG-neuron-specific neuropeptide gene *flp-17*. We dissected a 1460 bp *flp-17* promoter and found that a 138 bp promoter fragment was sufficient to drive expression in BAG neurons (Fig. S1). We noted that this minimal promoter contains three copies of a sequence motif predicted to bind ETS-family transcription factors. We then tested whether a promoter containing a single ETS-binding site is sufficient to drive gene expression in the BAG neurons. We observed that a 31 bp sequence containing a single ETS-binding site drove expression of a reporter transgene specifically in BAG neurons (Fig. 1A). These data indicated that one or more ETS-family transcription factors function to control BAG-cell fate specification.

Which of the ten ETS-family transcription factors encoded by the *C. elegans* genome regulates expression of BAG-neuron genes? We systematically analyzed the cell fate of CO_2_-chemosensitive BAG neurons in mutants for each ETS transcription factor. In wild-type animals, *Prom_flp-19_::gfp* is expressed by the two BAG neurons, and also by five other paired neurons [19] (Fig. 1 B,C). We found that nine ETS-gene mutants expressed the *Prom_flp-19_::gfp* reporter in BAG neurons. *ets-5* mutants, however, failed to express *Prom_flp-19_::gfp* in BAG neurons (Fig. 1B,C). Two independent *ets-*5 alleles caused the same defect in BAG neuron expression, and a fosmid containing the genomic *ets-5* locus complemented this defect. (Fig. 1C and Fig. S2).

**Figure 2:**
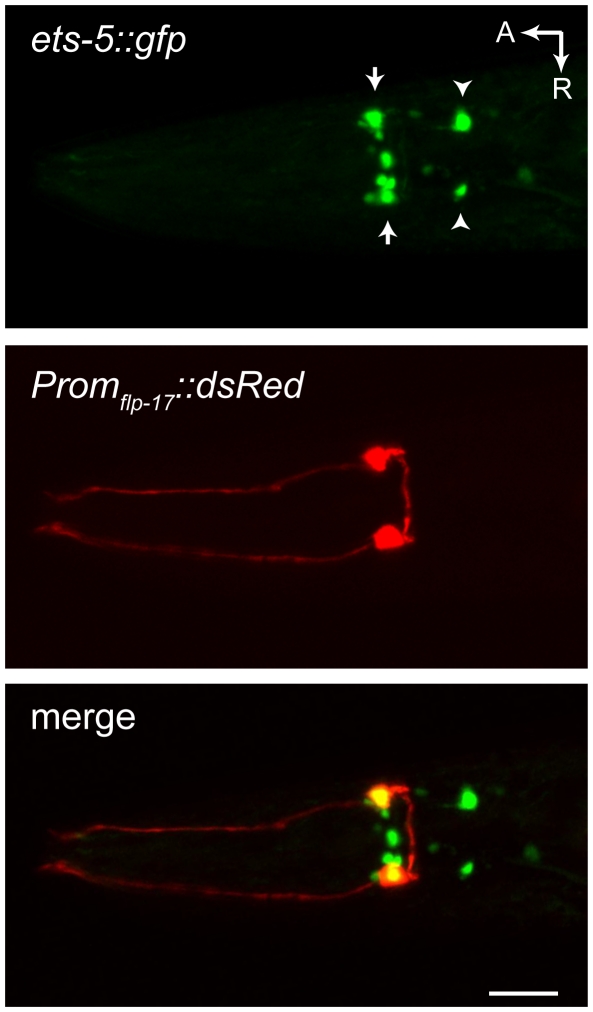
ETS-5 is expressed by a subset of head neurons, including the chemosensitive BAG neurons. Ventral view of an adult hermaphrodite expressing an *ets-5::gfp* translational reporter transgene. BAG neurons, which express a *Prom_flp-17_::dsRed* reporter, are indicated with arrows. Amphid neurons likely to be AWA neurons are indicated by arrowheads. A: anterior, R: right, the scale bar in lower panel is 20 µm. The *ets-5::gfp* transgene is *wzIs80* and the *Prom_flp-17_::dsRed* transgene is *wzEx36*.

These results confirm the recently reported role for ETS-5 in BAG neuron development [18]. We noted, however, an additional role for ETS-5 in the specification of AWA sensory neurons, which failed to express a *Prom_flp-19_::gfp* reporter transgene in *ets-5* mutants (Fig. 1B,C). A previous report indicated that *ets-5* is specifically expressed by the BAG neurons and suggested that ETS-5 functions only to specify BAG neurons but not other sensory neurons [18]. We determined the gene structure of *ets-5* by cloning an *ets-5* cDNA (Fig. S3), and we made an *ets-5::gfp* translational reporter transgene by inserting GFP coding sequences into the last exon of the *ets-5* genomic locus. We observed consistent expression of ETS-5::GFP in the nuclei of BAG neurons and fourteen other neuronal nuclei, including amphid neurons likely to be AWAs (Fig. 2). ETS-5 is therefore required for specification of BAG neurons, but neither its expression nor its function is restricted to BAG neurons.

### ETS-5 is required for sensory transduction in CO_2_-sensing BAG neurons

**Figure 3:**
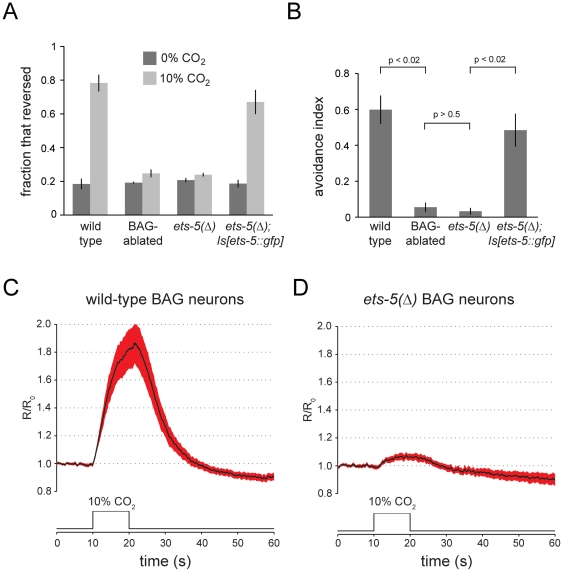
The BAG neurons of *ets-5* mutants are defective in sensory transduction. (A) *ets-5* mutants are defective in a BAG-neuron-dependent CO_2_ avoidance behavior. Plotted are the mean fractions of animals ± SEM that reversed during a four second exposure to either control atmosphere (0% CO_2_, 20% O_2_, balance N_2_) or CO_2_-enriched atmosphere (10% CO_2_, 20% O_2_, balance N_2_). Strains tested were: the wild-type strain N2, the BAG-ablated strain CX11697, the *ets-5* mutant strain FX1734, which carries the *tm1734* deletion allele of *ets-5*, and a derivative of FX1734 that carries the *ets-5::gfp* transgene *wzIs80*. *N* = 3–5 populations of 30–50 animals. (B) The effect of *ets-5* mutation on CO_2_ avoidance behavior is comparable to that of BAG neuron ablation. An avoidance index was calculated by subtracting the fraction of animals in a population that reversed in response to exposure to control atmosphere from the fraction that reversed in response to CO_2_-enriched atmosphere. Plotted are the mean avoidance indices for each of the four strains tested ± SEM. P values were calculated by one-way ANOVA. *N* = 3–5 populations of 30–50 animals. (C) Wild-type BAG neurons show robust calcium responses to a CO_2_ stimulus. Wild-type animals carrying a *Prom_gcy-9_::cameleon* transgene, which drives expression of cameleon specifically in BAG neurons, were immobilized and exposed to a 10 s pulse of 10% CO_2_. Plotted is the mean fractional ratio change in YFP/CFP emissions. The shaded area represents S.E.M. The cameleon expression transgene used was *wzIs82.* (D) The BAG neurons of *ets-5* mutants show reduced calcium responses to a CO_2_ stimulus. Animals carrying a variant *Prom_gcy-33_::cameleon* transgene, which drives *ets-5-*independent expression of cameleon in BAG neurons, were immobilized and exposed to a 10 s pulse of 10% CO_2_. Plotted is the mean fractional ratio change in YFP/CFP emissions. The shaded area represents S.E.M. The cameleon expression transgene used was *wzEx56*.

*ets-5* mutants are defective in an acute CO_2_ avoidance behavior [18], which we also observed (Fig. 3A). We monitored the locomotory behavior of animals exposed to a plume CO_2_-enriched artificial atmosphere and measured CO_2_-evoked reversals. The frequency of reversals by wild-type animals increased dramatically upon presentation of a CO_2_ stimulus (Fig. 3A). By contrast, the reversal frequency of animals carrying a caspase-expression transgene that specifically kills BAG neurons did not significantly change in response to presentation of a CO_2_ stimulus (Fig. 3A.) Like BAG-ablated animals, the reversal frequency of *ets-5* mutants did not significantly increase in response to a CO_2_ stimulus (Fig. 3A). The CO_2_-avoidance defect of *ets-5* mutants was complemented by the *ets-5::gfp* transgene used to determine its cellular expression pattern (Fig. 3A). *ets-5* is therefore necessary for acute CO_2_ avoidance, a behavior that is driven specifically by BAG neurons. The effect of CO_2_ on reversal frequencies of the different strains was calculated as an avoidance index, showing that BAG-ablated animals and *ets-5* mutants were comparably insensitive to CO_2_ (Fig. 3B).

To determine the effect of *ets-5* mutation on the physiology of BAG neurons, we used the ratiometric calcium indicator cameleon YC3.60 [20] to monitor the responses of wild-type and mutant BAG neurons to CO_2_ stimuli. Wild-type BAG neurons showed robust responses to CO_2_ stimuli; the mean fractional change in the ratio of YFP to CFP emissions to a 10% CO_2_ stimulus was greater than 80% (Fig. 3C). By contrast, *ets-5* mutant BAG neurons showed fractional ratio changes of less than 10% in response to the same CO_2_ stimulus (Fig. 3D). The BAG neurons of *ets-5* mutants are, therefore, defective in sensory transduction at a point upstream of the generation of an intracellular calcium transient. We next tested whether the transduction defect of *ets-5*-mutant BAG neurons might be caused by defects in the regulation of components of the CO_2_-transduction pathway.

### ETS-5 directly regulates the receptor-type guanylate cyclase gene *gcy-9*

**Figure 4:**
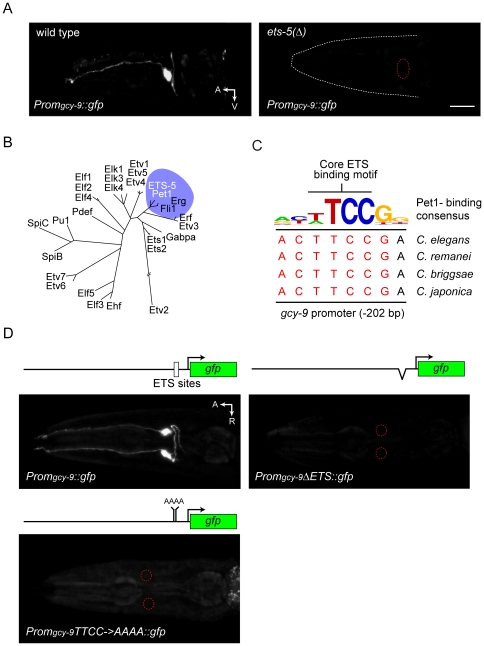
ETS-binding sites in the *gcy-9* promoter are required for its BAG-neuron expression. (A) Fluorescence micrographs of *Prom_gcy-9_::gfp* expression in a wild-type animal and an *ets-5* mutant. *Prom_gcy-9_::gfp* expression was principally in BAG neurons (top panel) and was lost in the *ets-5* mutant (bottom panel). In the lower panel, the BAGL neuron position is marked by a red circle. The scale bar in lower panel is 20 µm. A: anterior, V: ventral. The *ets-5* mutant allele was *tm1734*. The *Prom_gcy-9_::gfp* transgene was *wzEx37*. (B) The ETS domain of ETS-5 is most similar to that of mammalian Pet1. The ETS domains of ETS-5 and 26 mammalian ETS-family transcription factors were identified using the SMART database. Sequences were aligned using the CLUSTALW algorithm and sequence distances were plotted as a dendrogram using the Phylip sequence analysis package. (C) The *gcy-9* promoter contains an evolutionarily conserved ETS-binding motif that is highly similar to the Pet1-binding consensus. A logo of the Pet1-binding consensus sequence [24] is shown aligned to sequences approximately 200 bp upstream of *gcy-9* coding sequences from *C. elegans* and three other rhabditid nematodes. (D) ETS binding motifs in the *gcy-9* promoter are required for promoter function. Shown are ventral views of adult hermaphrodites carrying a 1.9 kb fragment of the *gcy-9* promoter fused to *gfp* coding sequences, or a promoter variant that either carries an 88 bp deletion centered around the ETS binding site at −202 bp, or a variant that carries four-base substitutions in the ETS-binding sites. In each case two lines were tested for GFP expression. Red circles indicate the location of BAG neurons in the animal carrying the mutant transgene. The wild-type *gcy-9* promoter transgene used was *wzEx37* and the mutant promoter transgene was *wzEx38*.

The receptor-type cyclase GCY-9 is a critical component of the CO_2_ transduction pathway (17), and a *Prom_gcy-9_*::*gfp* reporter transgene is not properly expressed by *ets-5* mutants (Fig. 4A). Does ETS-5 directly regulate *gcy-9*? We first sought to identify sequences in the *gcy-9* promoter that are required for expression by BAG neurons of a *Prom_gcy-9_*::*gfp* reporter. We noted that the ETS domain of ETS-5 is highly similar to the ETS domain of the vertebrate transcription factor Pet1 (Fig. 4B), which functions in the specification of neurons in the vertebrate midbrain [21], [22], [23]. Pet1 preferentially binds to a consensus sequence ACCGGAAGTA [24], and the *gcy-9* promoter contains a sequence that is almost identical to the reverse complement of this consensus sequence (Fig. 4C). Moreover, this putative ETS-binding site is highly conserved among nematode species, suggesting that it is functionally important (Figure 4C). This highly conserved ETS-site is adjacent to another putative ETS-binding site. We tested whether these presumptive ETS-binding sites are necessary for *gcy-9* promoter function by introducing into the *gcy-9* promoter a small deletion that removes them. We did not observe any activity of the mutant promoter in BAG neurons (Fig. 4D). We also introduced mutations in both ETS motifs, leaving the rest of the promoter intact. Like the deletion, these mutations abrogated expression of the reporter transgene, indicating that this motif is necessary for *gcy-9* promoter function.

**Figure 5:**
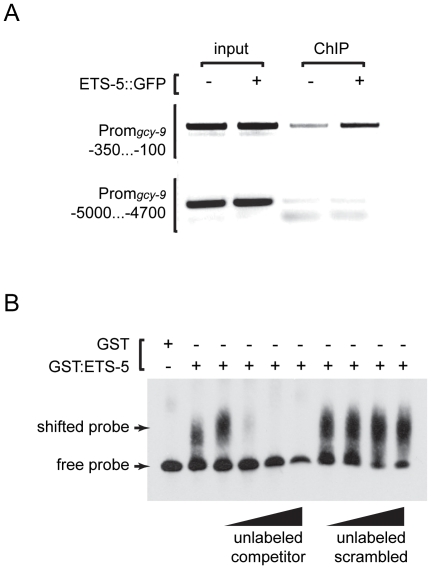
ETS-5 directly interacts with the *gcy-9* promoter. (A) ETS-5::GFP associates with the *gcy-9* promoter *in vivo*. Anti-GFP immunoprecipitates were prepared from cross-linked extracts of wild-type animals or animals carrying a functional *ets-5::gfp* transgene and interrogated for the presence of *gcy-9* promoter sequences by PCR. Immunoprecipitates from transgenic animals were enriched for *gcy-9* promoter sequences that contained the ETS-binding site at −202 bp. Control sequences at −5000 bp were not enriched in immunoprecipitates from transgenic animals. The *ets-5::gfp* transgene used was *wzIs80*. (B) ETS-5 binds to *gcy-9* promoter sequences *in vitro*. A mobility shift assay was performed with recombinant GST::ETS-5 and a 45 bp biotinylated DNA duplex probe containing the ETS-binding site from the *gcy-9* promoter. Recombinant GST::ETS-5 but not GST alone altered the electrophoretic mobility of the probe. The interaction between GST::ETS-5 and the probe was blocked by a molar excess of unlabeled probe but not by an excess of scrambled probe with the same nucleotide composition. Excess unlabeled wild-type and scrambled competitor probe was added in the following molar ratios: 10×, 50×, 100×, 500×.

We next tested whether ETS-5 associates *in vivo* with the *gcy-9* promoter at the ETS sites using chromatin immunoprecipitation. ETS-5::GFP immunoprecipitates were enriched for sequences containing the ETS binding site from the *gcy-9* promoter in comparison to precipitates prepared from extracts of non-transgenic animals, which did not express ETS-5::GFP (Fig. 5A). By contrast, sequences upstream of the ETS site were not significantly enriched in ETS-5::GFP immunoprecipitates. ETS-5, therefore, associates with a highly conserved and functionally important sequence element in the *gcy-9* promoter. To determine whether ETS-5 directly interacts with this element, we performed electrophoretic mobility shift assays (EMSAs) using a synthetic 45 base-pair DNA duplex containing the ETS binding site and recombinant ETS-5 protein (Fig. 5B). Recombinant ETS-5 altered the mobility of the duplex, and incorporation of labeled duplex into the complex was efficiently blocked by an excess of unlabeled duplex but not by sequence-scrambled duplex (Fig. 5B). Taken together with the observation that these ETS-binding motifs are required for the function of the *gcy-9* promoter (Fig. 4D), these data indicate that a direct interaction between ETS-5 and sequences in the *gcy-9* promoter is required for expression of *gcy-9* in BAG neurons.

### Expression of *gcy-9* bypasses a requirement for *ets-5* in CO_2_-avoidance

**Figure 6:**
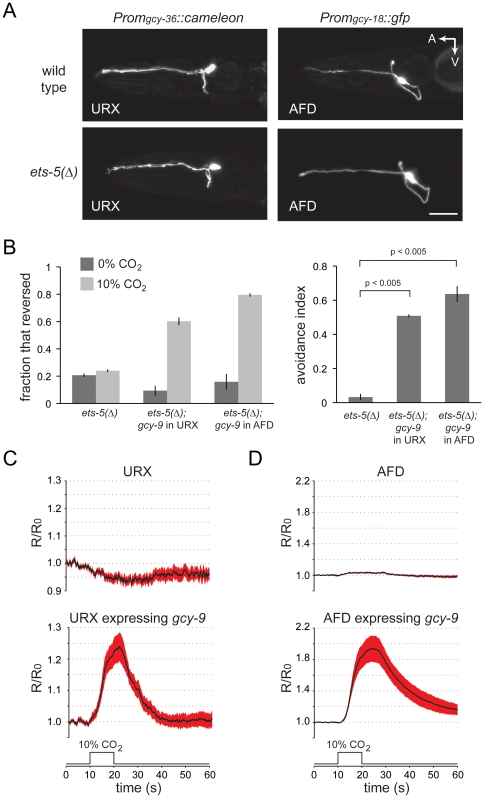
Expression of the ETS-5 target gene *gcy-9* restores CO_2_-chemosensitivity to *ets-5* mutants. (A) *gcy-36* and *gcy-18* promoters, which are active in oxygen-sensing and thermosensory neurons, respectively, are not regulated by ETS-5. Shown are lateral views of wild-type and *ets-5* mutant animals carrying either a *gcy-36* reporter, which is expressed by the oxygen-sensing URX neurons, or a *gcy-18* reporter, which is expressed by the thermosensory AFD neurons. A: anterior, V: ventral, scale bar is 20 µm. The *Prom_gcy-36_::cameleon* transgene used was *wzEx39* and the *Prom_gcy-18_::gfp* transgene was *wzEx40*. (B) Expression of *gcy-9* in either the URX oxygen sensors or the AFD thermosensors rescues the behavioral defect of *ets-5* mutants. The left plot shows the mean fraction of animals ± SEM that reversed during a four second exposure to either control atmosphere (0% CO_2_, 20% O_2_, balance N_2_) or CO_2_-enriched atmosphere (10% CO_2_, 20% O_2_, balance N_2_). The right plot shows the effect of CO_2_ on reversals as measured with an avoidance index, as in Fig. 2C. Plotted are the mean avoidance indices for each of the four strains tested ± SEM. P values were calculated by one-way ANOVA. *N* = 3–5 populations of 30–50 animals. The *ets-*5 mutant strain used was FX1734. The *Prom*_gcy*-36*_ *::gcy-9* transgene used was *wzIs97* and the *Prom_gcy-18_::gcy-9* transgene was *wzEx34*. (C) Expression of *gcy-9* in the URX oxygen sensors confers sensitivity to CO_2_. The ratiometric calcium indicator cameleon was expressed in the URX neurons. Wild-type URX neurons (top panel) showed a small decrease in the ratio of YFP:CFP emissions in response to 10 s CO_2_ pulses indicating decreases in cell calcium. URX neurons expressing *gcy-9* showed increases in cell calcium in response to CO_2_ stimuli, with an average ratio change of greater than 20% (lower panel). The cameleon expression transgene used was *wzIs96[Prom_gcy-32_::cameleon]* and the *gcy-9* expression transgene was *wzIs97[Prom_gcy-36_::gcy-9].* (D) Expression of *gcy-9* in the AFD thermosensors confers sensitivity to CO_2_. Calcium responses of wild-type AFD neurons (top panel) and AFD neurons that express *gcy-9* (bottom panel) in responses to a 10% CO_2_ stimulus. The cameleon expression transgene was *fxIs105[Prom_gcy-8_::cameleon]*, and the *Prom_gcy-18_::gcy-9* transgene was *wzEx34.* For panels C and D, plots are mean YFP/CFP emissions ratios normalized to the pre-stimulus ratio R_0_ (*N* = 16–22 animals). Red shaded areas represent SEM.

Are *ets-5* mutants defective in CO_2_ sensing principally because they fail to express *gcy-9*, or is *gcy-9* one of many BAG-cell-specific genes that are regulated by ETS-5 and necessary for CO_2_ chemosensation? To answer this question, we used promoters that are not regulated by ETS-5 to express *gcy-9* in neurons that (1) mediate an acute avoidance behavior similar to that triggered by BAG neurons and (2) like BAG neurons, use cGMP signaling. The *gcy-36* promoter is active in the URX oxygen-sensing neurons [25], which drive reversals in response to a hyperoxic stimulus [11]. The *gcy-18* promoter is specifically active in thermosensory AFD neurons [26], which also drive reversals [27]. Activity of neither the *gcy-18* nor the *gcy-36* promoter was affected by *ets-5* mutation (Fig. 6A). We then tested whether these promoters driving expression of *gcy-9* could rescue the CO_2_ avoidance defect of *ets-5* mutants. Unlike non-transgenic *ets-5* mutants, which failed to increase reversal frequency in response to a CO_2_ stimulus, *ets-5* mutants carrying either a *Prom_gcy-36_::gcy-9* transgene or a *Prom_gcy-18_::gcy-9* transgene showed robust avoidance responses to CO_2_ (Fig. 6B). A requirement for *ets-5* in CO_2_-avoidance behavior can therefore be bypassed by expressing the ETS-5 target gene *gcy-9* in neurons that are specified by *ets-5*-independent mechanisms.

Are URX and AFD neurons expressing the ETS-5 target gene *gcy-9* now CO_2_ chemosensors? We monitored the activity of wild-type and *gcy-9*-expressing URX and AFD neurons using *in vivo* calcium imaging. Wild-type URX neurons showed small decreases in cell calcium in response to a CO_2_ stimulus. By contrast, URX neurons of transgenic animals showed calcium increases that were phase-locked to the CO_2_ stimulus (Fig. 6C). We performed the same measurements on wild-type and transgenic AFD thermosensory neurons (Fig. 6D). Under some experimental conditions, AFD neurons demonstrate a calcium response to the falling edge of a CO_2_ pulse [14] *i.e.* an ‘off’ response. Under our experimental conditions, wild-type AFD neurons showed little response to CO_2_ stimuli (Fig. 6D). Transgenic AFD neurons expressing *gcy-9*, however, showed robust calcium responses to CO_2_ stimuli (Fig. 4D), demonstrating that expression of *gcy-9* in AFDs rendered them sensitive to CO_2_. Expression of the ETS-5 target gene, *gcy-9* is therefore sufficient to confer CO_2_ chemosensitivity upon neurons that do not normally respond to CO_2_.

## Discussion

Together, our data suggest that the mechanism by which ETS-5 determines CO_2_-sensitivity is to drive the expression of the receptor-type guanylate cyclase GCY-9, which likely functions as a receptor either for CO_2_ or for a CO_2_ metabolite such as bicarbonate or free protons. We previously showed that BAG neuron activation by CO_2_ requires cyclic-nucleotide-gated ion channels [17], suggesting that CO_2_-sensing by BAG neurons minimally requires a two-component transduction pathway: a CO_2_-activated guanylate cyclase and a cGMP-gated ion channel. Could such a transduction pathway exist in other species? Mammalian genomes encode receptor-type cyclases related to GCY-9. These cyclases have been matched to diverse ligands [28] and are also known to be regulated by interactions with anions, including chloride [29] and bicarbonate [30], [31], [32], [33]. Because vertebrate cyclases are modulated by the CO_2_ metabolite bicarbonate, this mechanism of cyclase activation might lie at the heart of an evolutionarily conserved pathway for CO_2_ detection.

Our data show that *gcy-9* expression is a determinant of whether a *C. elegans* neuron is CO_2_-chemosensitive. How is expression of *gcy-9* restricted to the BAG chemosensors? ETS-5 is necessary for expression of *gcy-9* but is also expressed by neurons that do not express *gcy-9* (Fig. 2). ETS-5 must therefore cooperate with additional, cell-type-specific factors to permit the activation of the *gcy-9* promoter specifically in BAG neurons. For example, overlapping expression of ETS-5 and a co-regulatory partner only in BAG neurons might drive cell-type-specific expression of GCY-9. Similar context-dependent neuronal specification events are known to require such ‘combinatorial coding’ [34]. Indeed, in other contexts, ETS-family transcription factors interact with co-regulatory transcription factors, including LIM-domain, homeodomain and JUN proteins [35], [36], [37]. Further studies will identify ETS-5 interacting partners that specifically drive expression of *gcy-9* in BAG neurons and consequently determine their CO_2_-chemosensitivity.

ETS-5 has the same domain structure as the vertebrate ETS protein Pet1, and its ETS domain, which mediates sequence-specific interactions with DNA [38], is almost identical to that of Pet1. Like ETS-5, Pet1 functions in the specification of neuronal cell fates [23]. Notably, among the neurons that are specified by Pet1 are serotonergic neurons, that are directly activated by CO_2_ and have recently been shown to function *in vivo* in the respiratory CO_2_ chemoreflex [21], [22]. In addition to promoting a serotonergic identity, Pet1, like ETS-5, might regulate the expression of genes that constitute a molecular pathway dedicated to the detection of CO_2_.

## Materials and Methods

### Strains used in this study

Strains used in this study are listed in Table S1. Strains were grown on NGM agar at 25°C on *Escherichia coli* OP50 except when used for chromatin immunoprecipitation experiments (see below). Transgenic animals were created according to Mello *et al.* [39]. *Prom_myo-2_::dsRed* coinjection marker was used at 5 ng ul^−1^. *Prom_myo-3_::dsRed* was used at 25 ng ul^−1^. *Prom_unc-122_::dsRed* was used at 30 ng ul^−1^. The *lin-15* rescuing plasmid pL15EK was used at 30 ng ul^−1^. *Prom_elt-2_::gfp* was used at 3 ng ul^−1^. *rol-6* was used at 100 ng ul^−1^. GFP, cameleon, dsRed and GCY-9 expression plasmids were injected at 100 ng ul^−1^. In some cases, extrachromosomal transgenes were integrated using gamma radiation (5000 rads).

### Plasmids

A complete list of plasmids used in this study is in Table S2.

### Microscopy

Young adults were anaesthetized with 30 mM sodium azide and mounted on a 2% agarose pad made in M9 medium. Series of confocal images were obtained with a Zeiss LSM510 microscope and maximum-projection images were created using ImageJ (Rasband, W.S., ImageJ, U. S. National Institutes of Health, Bethesda, Maryland, USA, http://imagej.nih.gov/ij/, 1997–2011).

### Behavioral Assay for Acute CO_2_ Avoidance

Behavioral assays were carried out essentially as described by Hallem and Sternberg [15]. Three or four trials of 30–50 animals were performed for each genotype. Animals were picked onto unseeded 6 cm nematode growth medium (NGM) agar plates. Gases were certified mixes of 20% oxygen with or without 10% CO_2_, balance nitrogen (Airgas).

### Statistical analysis

Standard errors and *P* values were calculated using GraphPad Prism analysis software. *P* values were calculated from a one-way ANOVA analysis of avoidance indexes.

### Chromatin immunoprecipitation

FQ237 *wzIs80[ets-5::gfp lin-15AB(+)]; lin-15AB(n765)* and N2 animals were grown on 10 cm peptone plates seeded with OP50. A mixed-stage culture of animals was repeatedly washed in M9 and harvested by centrifugation. 5 ml pellets of worms were resuspended in an equal volume of PBS+protease inhibitors and flash-frozen in liquid nitrogen. Frozen pellets were stored at −80°C prior to lysis.

To prepare extracts, frozen pellets were pulverized in a CryoCup (BioSpec) and cross-linked for 10 minutes by addition of 1.1% formaldehyde in PBS+PMSF. Formaldehyde was quenched by addition of 2.5 M glycine in PBS for 5 minutes. Cross-linked extracts were then washed in PBS, resuspended in FA buffer+0.1% sarkosyl, and sonicated using a Branson sonifier set to 35% output, 90% duty cycle with 8 sets of 12 pulses. Cross-linked sonicated extracts were then frozen at −80°C.

Prior to immunoprecipitation, extracts were thawed on ice. 5 µg of mouse anti-GFP IgG (Roche) was added to cross-linked sonicated extracts, incubated overnight at 4°C, and recovered using protein G agarose beads (Roche). Beads were washed as described by Ercan *et al.* [40] and immune complexes were eluted in 150 µl of 100 mM glycine pH 2.5. Protein-DNA crosslinks were reversed by incubation with proteinase K (Qiagen) at 65°C overnight. Nucleic acids were recovered using Qiaquick columns and eluted in 40 µl of elution buffer.

*gcy-9* promoter sequences were detected in immunoprecipitates using PCR. To detect the ETS site at −202 base pairs we used the primers 5′-GTTGCATTAGACAGTGTCATAG-3′ and 5′-CCGCAAACAGTAACAATTC-3′. To detect control sequences we used primers 5′-CAATGAGCTGTCCTGGCTTC- 3′ and 5′-TCGACATCTTTGACGCTGCTC-3′. 5 µl of sample from the chromatin immunoprecipitation was used as a template for a 30-cycle PCR.

### Electrophoretic mobility shift assay

Recombinant GST::ETS-5 was expressed in *E. coli* strain BL21and purified using glutathione sepharose affinity chromatography (GE Healthcare). 1 µg of recombinant ETS-5 or 2 µg of GST was added to each reaction, which contained 0.1 pmol of 3′-biotin-labeled duplex probe**.** In some reactions, a molar excess of unlabeled competitor duplex (identical or sequence-scrambled) was included. Reactions were incubated at room temperature for 20 minutes and protein-DNA complexes were separated from unbound probe by electrophoresis in a 6% polyacrylamide gel in 0.5× TBE. Nucleic acids were then transferred to a nylon membrane (Amersham, GE Healthcare) and detected using HRP-coupled streptavidin and enhanced chemiluminscence (Pierce Protein Research Products).

The sequence of the biotinylated probe and the unlabeled competitor was:

5′-ATCCACTTCCGATGGGCCCTTCCGGCATGATAAGAAGTGAGTGGC-3′.

The sequence of the scrambled competitor was:

5′-CTGACCCCGTGTCGCTAATAGACTCAGTTACGCAAGATGG-3′.

### *in vivo* calcium imaging

Calcium imaging was performed essentially as previously described [17]. Young adults were immobilized with cyanoacrylate veterinary glue (Surgi-Lock; Meridian Animal Health) on a cover glass coated with a 2% agarose pad made with 10 mM HEPES (pH 7.4). The cover glass was affixed to a custom-made air chamber. The specimen was illuminated with 435-nm excitation light and imaged using a 40× Nikon long-working distance objective (0.75 numerical aperture). The emission image was passed through a DV2 image splitter (Photometrics), and the CFP and YFP emission images were projected onto two halves of a cooled CCD camera (Andor). Images were acquired at 10 Hz, with exposure times between 10 and 50 ms. Gas perfusion was controlled by three-way valves (Numatics) driven by a custom-made valve controller unit. Excitation light, image acquisition, and hardware control were performed by the Live Acquisition software package (Till Photonics). Post-acquisition analysis of ratio plots was performed using custom Matlab scripts, which subtracted linear baseline drift from traces and applied a five-frame boxcar filter to the ratio time series. Custom certified gas mixes used for imaging were obtained from Airgas.

## Supporting Information

Figure S1 **Deletion analysis of the** ***flp-17*** **promoter.** (A) Genomic locus of the *flp-17* gene with the location of predicted ETS binding sites shown as blue bars. mCherry protein was driven by the promoter elements indicated with black bars and expression in the BAG neurons was scored. The *Prom9_flp-17_* is a 138 bp fragment that is sufficient to drive expression of mCherry protein in the BAG neurons. *N*≥30 animals/transgenic line. (B) Dorsal view of *Prom9_flp-17_::mCherry* expression in a wild-type animal. BAG neuron positions are marked by red circles. The scale bar is 20 µm.
(TIF)

Figure S2 ***ets-5*** **mutants have defects in expression of multiple BAG-neuron genes.** (A) An independently derived allele of *ets-5* has BAG neurons that fail to express a *Prom_flp-19_::gfp* transgene. The fraction of transgenic *ets-5(tm866)* and *ets-5(tm1734)* animals that express GFP in BAG neurons is plotted next to the wild type (+) and *ets-5(tm1734)* mutants carrying rescuing transgenes derived from a fosmid that encompasses the *ets-5* locus. *N*≥30, # = independent transgenic lines. (B) Dorsal views of *Prom_flp-13_::gfp* expression in a wild-type animal and an *ets-5* mutant. *Prom_flp-13_::gfp* expression was lost in the *ets-5* mutant (bottom panel). BAG neuron positions are marked by red circles. The scale bar in lower panel is 20 µm. A: anterior, V: ventral. The *ets-5* mutant allele was *tm1734*. The *Prom_flp-13_::gfp* transgene was *ynIs37.* (C) Lateral views of *Prom_flp-17_::gfp* expression in a wild-type animal and an *ets-5* mutant. *Prom_flp-17_::gfp* expression was lost in the *ets-5* mutant (bottom panel). BAG neuron position in the lower panel is marked by a red circle. The scale bar in lower panel is 20 µm. A: anterior, V: ventral. The *ets-5* mutant allele was *tm1734*. The *Prom_gcy-17_::gfp* transgene was *ynIs64*. (D) Lateral views of *Prom_gcy-31_::gfp* expression in a wild-type animal and an *ets-5* mutant. *Prom_gcy-31_::gfp* expression was lost in the *ets-5* mutant (bottom panel). BAG neuron position in the lower panel is marked by a red circle. The scale bar in lower panel is 20 µm. A: anterior, V: ventral. The *ets-5* mutant allele was *tm1734*. The *Prom_gcy-17_::gfp* transgene was *ynIs64*.
(TIF)

Figure S3 **Characterization of the** ***ets-5*** **transcript.** (A) Genomic organization of *ets-5* coding sequences as determined by amplification of *ets-5* cDNA using gene-specific and SL1 primers. cDNAs derived from SL1 trans-spliced messages contained 95 bases of 5′ non-coding sequence (white box) and were organized into five exons. Brackets indicate regions deleted by the *tm1734* and *tm866* mutations. (B) Nucleotide sequence of *ets-5* cDNA. The SL1 leader sequence is underlined. Non-coding sequences are lowercase and exons are denoted by text boxes alternately colored blue and green. (C) Predicted amino acid sequence of ETS-5. Sequences of the ETS homology domain are in white text on a black background.
(TIF)

Table S1 **Strains used in this study.** Strain designations and complete genotypes of all the strains used in this study.
(DOC)

Table S2 **Plasmids used in this study.** Complete descriptions of plasmids used in this studies and the sequences of primers used for their construction.
(DOC)
