# Supplementary figures and images for: A Single Gene Target of an ETS-Family Transcription Factor Determines Neuronal CO2-Chemosensitivity
Source: PLoS One. 2012 Mar 29;7(3):e34014. doi: 10.1371/journal.pone.0034014 (PMC3315506; doi:10.1371/journal.pone.0034014)

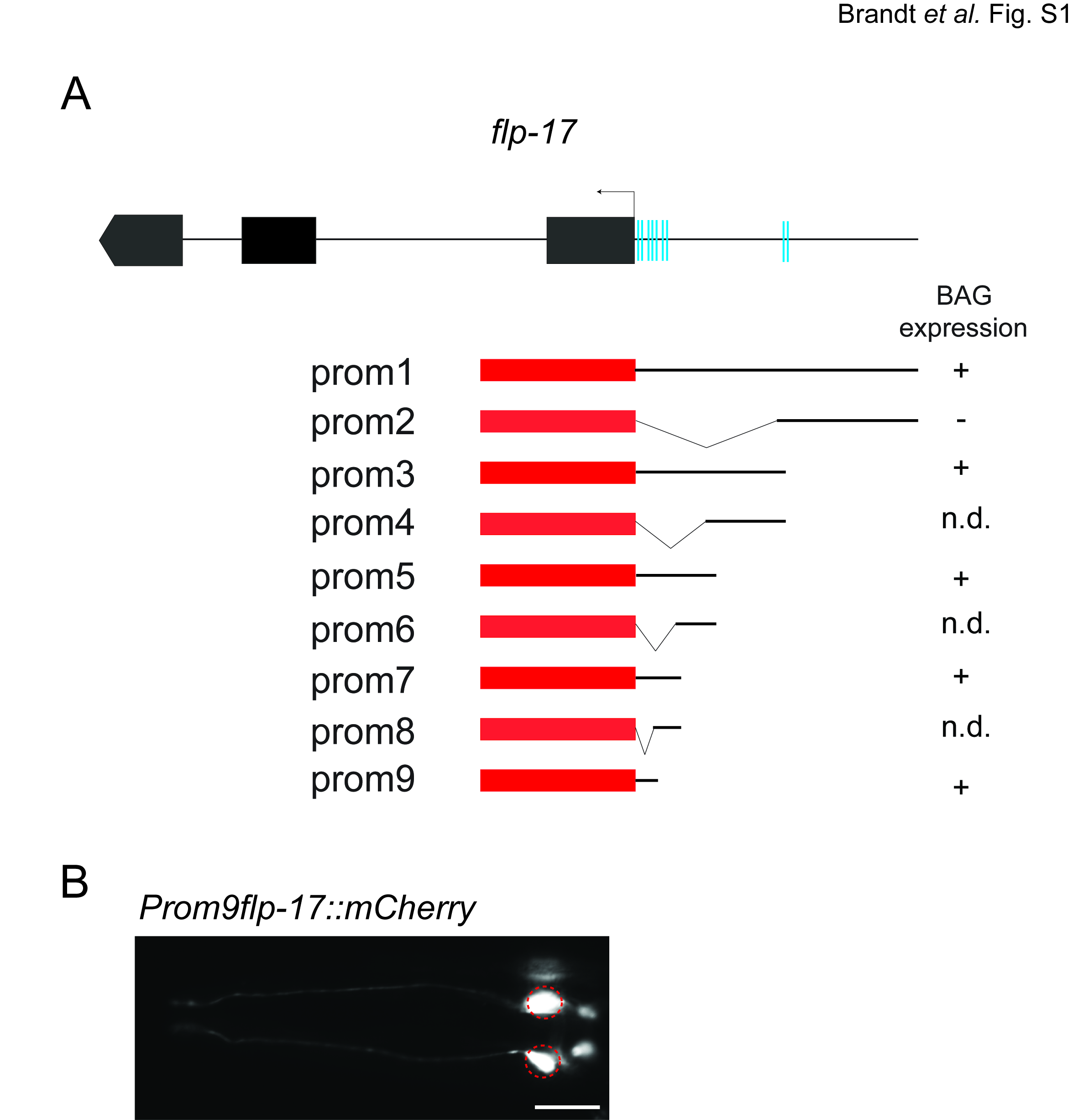

Supplement: Figure S1 — Deletion analysis of the flp-17 promoter. (A) Genomic locus of the flp-17 gene with the location of predicted ETS binding sites shown as blue bars. mCherry protein was driven by the promoter elements indicated with black bars and expression in the BAG neurons was scored. The Prom9flp-17 is a 138 bp fragment that is sufficient to drive expression of mCherry protein in the BAG neurons. N≥30 animals/transgenic line. (B) Dorsal view of Prom9flp-17::mCherry expression in a wild-type animal. BAG neuron positions are marked by red circles. The scale bar is 20 µm. (TIF) [file pone.0034014.s001.tif]

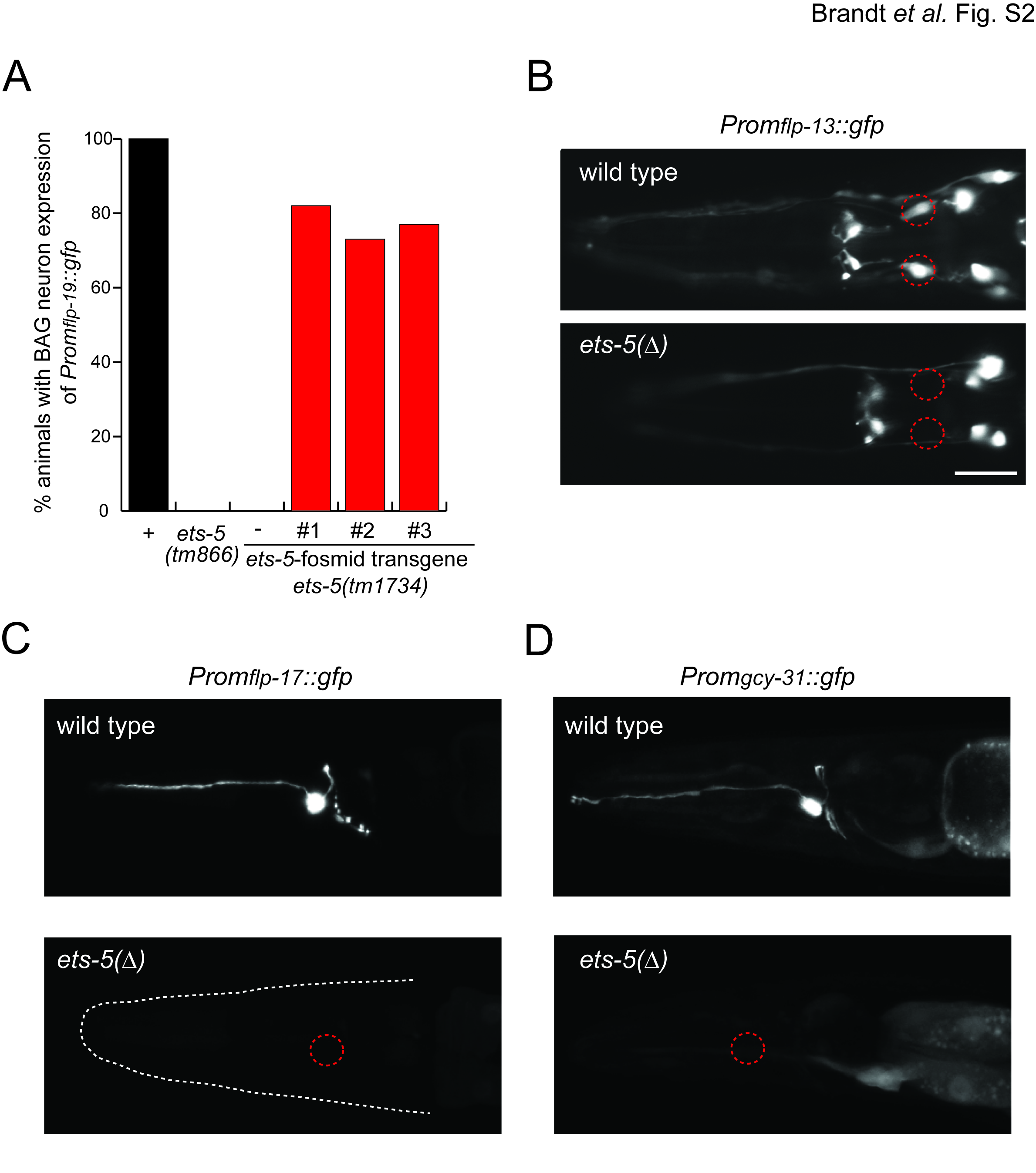

Supplement: Figure S2 — ets-5 mutants have defects in expression of multiple BAG-neuron genes. (A) An independently derived allele of ets-5 has BAG neurons that fail to express a Promflp-19::gfp transgene. The fraction of transgenic ets-5(tm866) and ets-5(tm1734) animals that express GFP in BAG neurons is plotted next to the wild type (+) and ets-5(tm1734) mutants carrying rescuing transgenes derived from a fosmid that encompasses the ets-5 locus. N≥30, # = independent transgenic lines. (B) Dorsal views of Promflp-13::gfp expression in a wild-type animal and an ets-5 mutant. Promflp-13::gfp expression was lost in the ets-5 mutant (bottom panel). BAG neuron positions are marked by red circles. The scale bar in lower panel is 20 µm. A: anterior, V: ventral. The ets-5 mutant allele was tm1734. The Promflp-13::gfp transgene was ynIs37. (C) Lateral views of Promflp-17::gfp expression in a wild-type animal and an ets-5 mutant. Promflp-17::gfp expression was lost in the ets-5 mutant (bottom panel). BAG neuron position in the lower panel is marked by a red circle. The scale bar in lower panel is 20 µm. A: anterior, V: ventral. The ets-5 mutant allele was tm1734. The Promgcy-17::gfp transgene was ynIs64. (D) Lateral views of Promgcy-31::gfp expression in a wild-type animal and an ets-5 mutant. Promgcy-31::gfp expression was lost in the ets-5 mutant (bottom panel). BAG neuron position in the lower panel is marked by a red circle. The scale bar in lower panel is 20 µm. A: anterior, V: ventral. The ets-5 mutant allele was tm1734. The Promgcy-17::gfp transgene was ynIs64. (TIF) [file pone.0034014.s002.tif]

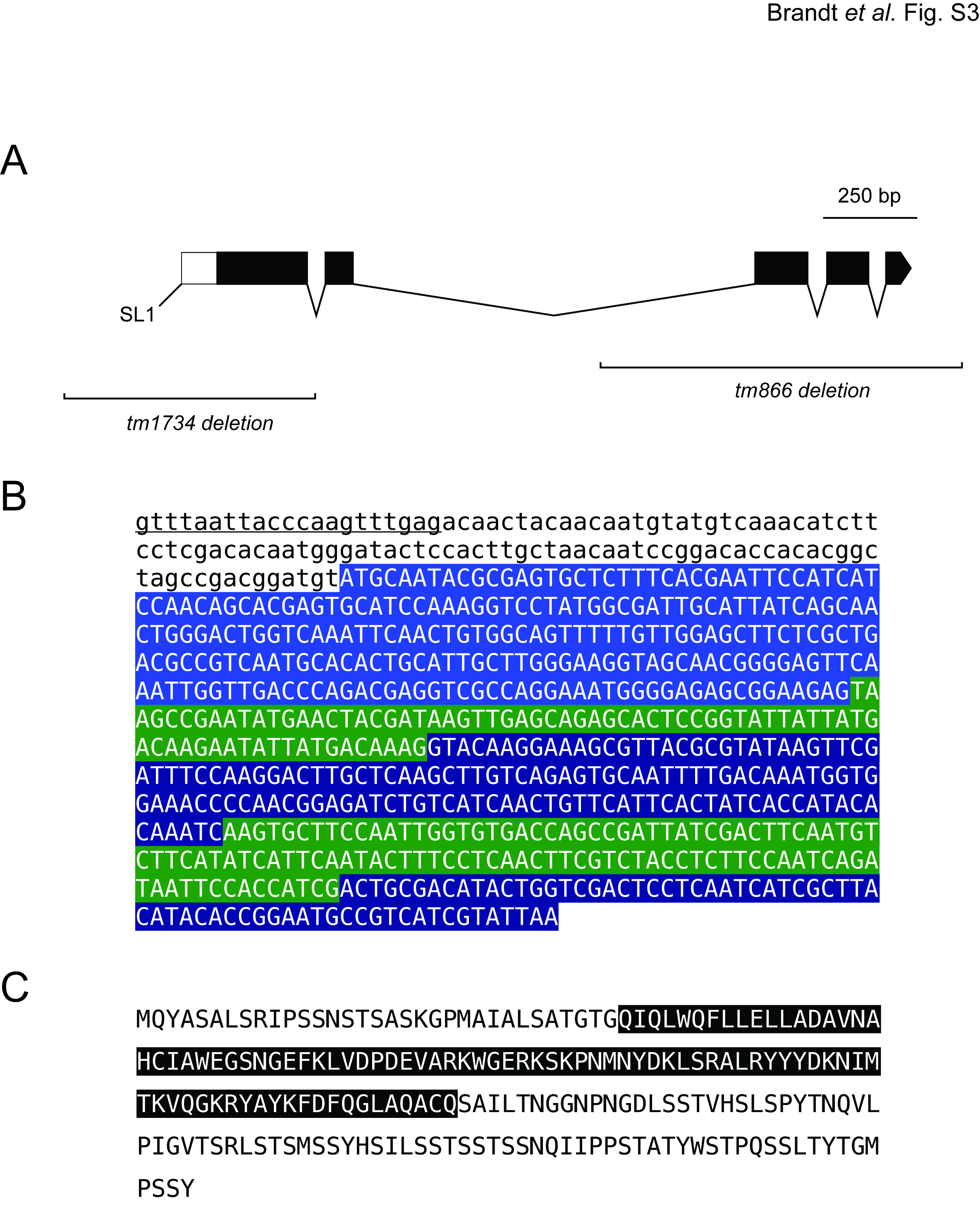

Supplement: Figure S3 — Characterization of the ets-5 transcript. (A) Genomic organization of ets-5 coding sequences as determined by amplification of ets-5 cDNA using gene-specific and SL1 primers. cDNAs derived from SL1 trans-spliced messages contained 95 bases of 5′ non-coding sequence (white box) and were organized into five exons. Brackets indicate regions deleted by the tm1734 and tm866 mutations. (B) Nucleotide sequence of ets-5 cDNA. The SL1 leader sequence is underlined. Non-coding sequences are lowercase and exons are denoted by text boxes alternately colored blue and green. (C) Predicted amino acid sequence of ETS-5. Sequences of the ETS homology domain are in white text on a black background. (TIF) [file pone.0034014.s003.tif]
