# Supplementary material for: A Single Gene Target of an ETS-Family Transcription Factor Determines Neuronal CO2-Chemosensitivity
Source: PLoS One. 2012 Mar 29;7(3):e34014. doi: 10.1371/journal.pone.0034014 (PMC3315506; doi:10.1371/journal.pone.0034014)
Supplement: Table S1 — Strains used in this study. Strain designations and complete genotypes of all the strains used in this study. (DOC) [file pone.0034014.s004.doc]

**Table S1. Strains used in this study**

**STRAIN GENOTYPE**

RJP255 *ynIs34[Prom_flp-19_::gfp]*

RJP248 *ets-5(tm1734); ynIs34[Prom_flp-19_::gfp]*

RJP259 *ets-5(tm866); ynIs34[Prom_flp-19_::gfp]*

RJP258 *ast-1(hd1); ynIs34[Prom_flp-19_::gfp]*

RJP98 *C50A2.4(tm440); ynIs34[Prom_flp-19_::gfp]*

RJP246 *lin-1(e1777); ynIs34[Prom_flp-19_::gfp]*

RJP236 *C52B9.2(tm413);* *ynIs34[Prom_flp-19_::gfp]*

RJP234 *F19F10.1(tm456);* *ynIs34[Prom_flp-19_::gfp]*

RJP257 *C24A1.2(tm801);* *ynIs34[Prom_flp-19_::gfp]*

RJP318  *F19F10.5(ok888);* *ynIs34[Prom_flp-19_::gfp]*

RJP237 *ets-4(ok165);* *ynIs34[Prom_flp-19_::gfp]*

RJP238 *tag-97(ok286);* *ynIs34[Prom_flp-19_::gfp]*

RJP549 *ets-5(tm1734);* *ynIs34[Prom_flp-19_::gfp]; rpEx246[ets-5 fosmid myo‑3::mCherry]*

FQ329 *lin-15AB(n765); wzEx37[Prom_gcy-9_::gfp lin-15(+)]*

FQ336 *ets-5(tm1734) lin-15AB(n765); wzEx37[Prom_gcy-9_::GFP lin-15(+)]*

FQ237  *wzIs80[ets-5::gfp lin-15(+)]; lin-15AB(n765)*

FQ341  *wzIs80[ets-5::gfp lin-15AB(+)]; lin-15AB(n765); wzEx36[Prom_flp‑17_::dsRed]*

CX11697 *kyIs536[Prom_flp-17_::caspase p17::SL2::gfp Prom_elt-2_::gfp]*

*kyIs538[Prom_glb-5_::caspase p12::SL2::gfp Prom_elt-2_::dsRed]*

FX1734 *ets-5(tm1734)*

FQ304 *wzIs80[ets-5::gfp lin-15(+)]; lin-15AB(n765); ets-5(tm1734) lin‑15AB(n765)*

*FQ388 wzEx60[ Prom_gcy-33_::cameleon Prom_myo-2_::dsRed]*

FQ334 *lin-15AB(n765); wzEx38 [Prom_gcy-9∆ets_::gfp]*

*FQ386 wzEx58 [Prom_gcy-9TTCC->AAAA_::gfp Prom_unc-122_::mCherry]*

FQ325  *lin-15AB(n765); wzEx40[Prom_gcy-18_::gfp]*

FQ331 *ets-5(tm1734); wzEx40[Prom_gcy-18_::gfp]*

FQ332 *wzEx39[Prom_gcy-36_::CAM Prom_gcy-35_::gcy-9]*

FQ324 *ets-5(tm1734); wzEx39[Prom_gcy-36_::cameleon Prom_gcy-35_::gcy-9]*

FQ320 *ets-5(tm1734); wzEx34[gcy-18::gcy-9 Prom_myo-2_::dsRed]*

FQ330 *wzIs97[Prom_gcy-36_::gcy-9 Prom_myo-3_::dsRed]; ets-5(tm1734)*

PX433 *fxIs105[Prom_gcy-8_::cameleon Prom_unc122_::dsRed]*

FQ323 *wzIs96[Prom_gcy-32_::cameleon Prom_unc-122_::dsRed]*

FQ335 *wzIs97[Prom_gcy-36_::gcy-9 Prom_myo-3_::dsRed]*

FQ301 *fxIs105[Prom_gcy-8_::cameleon Prom_unc-122_::dsRed]; wzEx34[Prom_gcy‑18_::gcy-9 Prom_myo-2_::dsRed]*

**Strains used in supplemental figures**

RJP43-45 *rpEx10, 11, 12[Prom1_flp-17_::mCherry]*

RJP222-224 *rpEx64, 65, 66[Prom2_flp-17_::mCherry]*

RJP263 *rpEx85[Prom3_flp-17_::mCherry]*

RJP509-10 *rpEx227, 228[Prom5_flp-17_::mCherry]*

RJP511-13 *rpEx229-231[Prom7_flp-17_::mCherry]*

RJP536-38 *rpEx239-241[Prom9_flp-17_::mCherry]*

RJP550 *ets-5(tm1734); ynIs34[Prom_flp-19_::gfp]; rpEx247[ets-5 fosmid myo‑3::mCherry]* #2

RJP551 *ets-5(tm1734); ynIs34[Prom_flp-19_::gfp]; rpEx248[ets-5 fosmid myo‑3::mCherry]* #3

NY1037 *ynIs37[Prom_flp-13_::GFP]*

RJP517 *ets-5(tm1734); ynIs37[Prom_flp-13_::GFP]*

NY2064 *ynIs64[Prom_flp-17_::GFP]*

FQ223  *ynIs64[Prom_flp-17_::GFP]; ets-5(tm1734)*

CX6983 *kyEx2116 [Prom_gcy-31_::SL2::GFP odr-1::dsRed2]*

FQ256 *kyEx2116 [Prom_gcy-31_::SL2::GFP odr-1::dsRed2]; ets-5(tm1734)*
