# Supplementary material for: A Single Gene Target of an ETS-Family Transcription Factor Determines Neuronal CO2-Chemosensitivity
Source: PLoS One. 2012 Mar 29;7(3):e34014. doi: 10.1371/journal.pone.0034014 (PMC3315506; doi:10.1371/journal.pone.0034014)
Supplement: Table S2 — Plasmids used in this study. Complete descriptions of plasmids used in this studies and the sequences of primers used for their construction. (DOC) [file pone.0034014.s005.doc]

**Table S2. Plasmids used in this study**

***Prom_31bp_::gfp* reporter.**

The synthetic 31bp element containing an ETS binding site was made by annealing the following oligos:

5’‑AGCTTgatgatttgatacGGAAGGAAatgtggtgtcG-3’ and 5’‑GATCCGACACCACATTTCCTTCCGTATCAAATCATCA -3’.

The annealed oligos were ligated into the vector pPD95.75 between restriction sites *Hind*III and *BamH*I*.*

***Prom_gcy-9_::gfp* reporter.** The *gcy-9* promoter was amplified using primers

NR198 5’‑GCCTGGCAGATGCGAGGAAAACTAATGGAGGCG-3’ and

NR199 5’‑ACCGGATCCAATGAAAAATAAATATAAACGCAT-3’.

The amplicon was ligated into the vector pPD95.77 between restriction sites *Pst*I and *BamH*I producing the plasmid pNR395.

***ets-5::gfp* translational reporter.** The *ets-5* genomic locus was amplified by PCR using the primers

SAZ6 5’‑GACTCTGCAGTCGCAACATTCAATGGAGCCTGC-3’ and

NR271 5’‑TACCGGTACCTTATACGATGACGGCATTCCGGTG-3’.

The cloned PCR product was sequenced and subcloned into the vector pPD95.77 using *Pst*I and *Kpn*I restriction sites, generating the plasmid pSAZ41.

***Prom_flp-17_:rfp* reporter.** Amplification of 3.3kb upstream of the start site of *flp-17* annotated in *ynIs64* was carried out with the primers

JKL3 5’-GATCAGGGATCCCTGGAAAAATAAAGTTTTGCG-3’ and

JKL4 5’-GATCAGCTGCAGCCTTGAAGCTTTTCCTCTG-3’.

The amplicon was ligated in a dsRed variant of the Fire vector pPD95.77 using the restriction sites *Bam*HI and *Pst*I, to produce plasmid pJB134.

***Prom_gcy-9ΔETS_::gfp* reporter.** 88 base pairs centered around -202 base pairs upstream of the *gcy-9* start site were excised from pNR395 by inverse PCR using the primers NR355 5’-AGATGGGGTTTAATGGAAAGAAGG-3’ and

NR356 5’-CATGATAAGAAGTGAGTGGCTAC-3’.

The resulting plasmid was pJB102.

***Prom_gcy-9_*** ***_TTCC->AAAA_::gfp* reporter.** Using the Stratagene Quik Change II Site Directed Mutagenesis kit, point mutations in the two ETS sites deleted in pJB102 were introduced. The first ETS site contained in the 88 base pair deletion was mutated using the primers

JB88 5’- CTTCCGATGGGCCCTTTTTTCATGATAAGAAGTGAG-3’ and

JB89 5’- CTCACTTCTTATCATGAAAAAAGGGCCCATCGGAAG-3’.

This yielded the plasmid pJB171, which was re-mutagenised at the second ETS site using the primers

JB90 5’- CAAAAGATGGGCCCAAAAGGCATGATAAGAAGTGAG-3’ and

JB91 5’- CTCACTTCTTATCATGCCTTTTGGGCCCATCTTTTG-3’.

The resulting plasmid pJB172 was mutated at both ETS sites from TTCC to AAAA.

**Recombinant GST-ETS-5.** *ets-5* cDNA for the GST fusion protein was amplified using the primers

SAZ63 5’-GGATCCATGCAATACGCGAGTGCTCTTTCA-3’ and

SAZ64 5’-CTCGAGTTAATACGATGACGGCATTCCGGT-3’.

The amplicon was cloned into the PGEX vector using restriction sites *BamH*I and *Xho*I to generate the plasmid pSAZ148.

***Prom_gcy-18_::gfp* reporter.** The *gcy-18* promoter was amplified using the primers

SAZ15 5’-GACTCTGCAGTCGATGCAACTGAGCTCCACTGC-3’ and

SAZ16 5’-GACTGGATCCTTTCTGATGCTCCGACTGCAAAGTAGC-3’.

The amplicon was ligated into pPD95.77 between restriction sites *Pst*I and *Bam*HI, generating the plasmid pSAZ138.

***Prom_gcy-36_::cameleon* reporter.** The *gcy-36* promoter was amplified using primers NR352 5’-GCATCTGCAGTTGGCATGTATTCTCGGCATTTTGC-3’ and

NR353 5’-GCATGGATCCTGTTGGGTAGCCCTTGTTTGAATTTACC-3’.

The amplicon was ligated into PCVG6 cameleon vector using *Sbf*I and *Bam*HI sites, producing the plasmid pSAZ119.

***Prom_gcy-35_::gcy-9* expression construct.** The *gcy-35* promoter was amplified using primers

NR350 5’-GCACCTGCAGGATGGCGGTTTGAACCTCCGCCGAGG-3’ and

NR351 5’-CGAGGATCCATTCTACTCTCCGCAAAAAAGTAACG-3’.

The amplicon was ligated into the first multiple cloning site of pPD49.26 using restriction sites *Sbf*I and *BamH*I*.* *gcy-9* cDNA was amplified using the primers

NR239 5’-GACTGCATAGCAGAAAAATGCGTTTATATTTATTTTTCATTTC-3’ and

NR240 5’- GACTGGTACCTCATTGTTTGCCGGTTCTTCCTTC-3’.

The *gcy-9* cDNA was cloned into the second multiple cloning site of pPD49.26 using *Nhe*I and *Kpn*I to generate the plasmid pSAZ109.

***gcy-9* ectopic expression constructs.** The promoters of *gcy-18* and *gcy-*36 (described above) were subcloned into the first multiple cloning site of a pPD49.26 variant that contained *gcy-9* cDNA in the second multiple cloning site. *Prom_gcy-18_::gcy-9* ectopic expression construct plasmid was pSAZ30. *Prom_gcy-36_::gcy-9* ectopic expression construct plasmid was pSAZ115.

***Prom_gcy-32_::cameleon* reporter.** The *gcy-32* promoter was amplified using the primers LAM11 5’- CCTGCAGGCTCAGTTTTCCAAAGGCGAAACGT-3’ and

LAM12 5’-GGATCCTCTATAATACAATCGTGATCTTCGCTTCGG-3’.

The PCR product was subcloned into the cameleon vector pCVG6 between restriction sites *Sbf*I and *Bam*HI*.*

**Plasmids used in Supplemental Figures**

**Cloning of *ets-5* cDNA**

To clone the 5’ end of cDNAs of *ets-5* transcripts, nested PCR was performed using a forward primer that matched the sequence of the SL1 trans-spliced leader and reverse primers complementary to highly conserved sequences in the predicted transcript: NR275 5’-GCACTCTGACAAGCTTGAGCAAGTCC-3’

NR276 5’-TCGAACTTATACGCGTAACGCTTTCC-3’

PCR products were cloned into the vector pGEMTeasy (Promega) and sequenced.

**Promoter fusions used in Supplemental Figures**

***Prom1_flp17_::mCherry* fusion.** The *flp-17* promoter1 (1461bp) was amplified and fused to the pPD95.75 mCherry using primers :

PCR1: oVJ-9 5’- attgttttagtttgatagatcgg-3’ and oVJ10 5’- AGTCGACCTGCAGGCATGCAAGCTctggaaaaataaagttttgcg-3’

PCR2: oVJ11 5’- cattttttcatgaaaatt-3’ and

and D*5’- GGAAACAGTTATGTTTGGTATA-3’

***Prom2_flp17_::mCherry* fusion.** The *flp-17* promoter2 (676bp) was amplified using primers:

PCR1: oVJ-9-5’-attgttttagtttgatagatcgg-3’ and oVJ20 5’- AGTCGACCTGCAGGCATGCAAGCTttcgcaaactccgtgatatttcg-3

PCR2: oVJ11 5’- cattttttcatgaaaatt-3’ and

and D*5’- GGAAACAGTTATGTTTGGTATA-3’

***Prom3_flp17_::mCherry* fusion.** The *flp-17* promoter3 (785bp) was amplified using primers:

PCR1: oVJ-21 5’-aatatcacggagtttgcg-3’ and oVJ10 5’- AGTCGACCTGCAGGCATGCAAGCTctggaaaaataaagttttgcg-3’

PCR2: oVJ22 5’- aaatggagcgggcttgaac-3’ and D*5’- GGAAACAGTTATGTTTGGTATA-3’

***Prom5_flp17_::mCherry* fusion.** The *flp-17* promoter5 (450bp) was amplified and fused to the pPD95.75 mCherry using primers :

PCR1: oKL-34 5’-gcagtaggcatgtggtag-3’ and oVJ10 5’- AGTCGACCTGCAGGCATGCAAGCTctggaaaaataaagttttgcg-3’

PCR2: oKL-35 5’-gcatgtggtaggcaagc-3’ and D*5’- GGAAACAGTTATGTTTGGTATA-3’

***Prom7_flp17_::mCherry* fusion.** The *flp-17* promoter7 (195bp) was amplified and fused to the pPD95.75 mCherry using primers:

PCR1: oVJ3 5’-cagtgatttcaatcggaaattc-3’ and oVJ10 5’- AGTCGACCTGCAGGCATGCAAGCTctggaaaaataaagttttgcg-3’

PCR2: oVJ2 5’-caatcggaaattcggagcc-3’ and D*5’- GGAAACAGTTATGTTTGGTATA-3’

***Prom9_flp17_::mCherry* fusion.** The *flp-17* promoter9 (138bp) was amplified and fused to the pPD95.75 mCherry using primers:

PCR1: oVJ3 5’-cagtgatttcaatcggaaattc-3’ and oVJ10 5’- AGTCGACCTGCAGGCATGCAAGCTctggaaaaataaagttttgcg-3’

PCR2: oVJ6 5’-cacgggaaattcagatttttc-3’ and D*5’- GGAAACAGTTATGTTTGGTATA-3’
